# Effects of Temporal Resolution on an Inferential Model of Animal Movement

**DOI:** 10.1371/journal.pone.0057640

**Published:** 2013-05-06

**Authors:** Claire M. Postlethwaite, Todd E. Dennis

**Affiliations:** 1 Department of Mathematics, University of Auckland, Auckland, New Zealand; 2 School of Biological Sciences, University of Auckland, Auckland, New Zealand; Institut Pluridisciplinaire Hubert Curien, France

## Abstract

Recently, there has been much interest in describing the behaviour of animals by fitting various movement models to tracking data. Despite this interest, little is known about how the temporal ‘grain’ of movement trajectories affects the outputs of such models, and how behaviours classified at one timescale may differ from those classified at other scales. Here, we present a study in which random-walk state-space models were fit both to nightly geospatial lifelines of common brushtail possums (*Trichosurus vulpecula*) and synthetic trajectories parameterised from empirical observations. Observed trajectories recorded by GPS collars at 5-min intervals were sub-sampled at periods varying between 10 and 60 min, to approximate the effect of collecting data at lower sampling frequencies. Markov-Chain Monte-Carlo fitting techniques, using information about movement rates and turning angles between sequential fixes, were employed using a Bayesian framework to assign distinct behavioural states to individual location estimates. We found that in trajectories with higher temporal granularities behaviours could be clearly differentiated into ‘slow-area-restricted’ and ‘fast-transiting’ states, but for trajectories with longer inter-fix intervals this distinction was markedly less obvious. Specifically, turning-angle distributions varied from being highly peaked around either 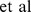 or 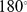 at fine temporal scales, to being uniform across all angles at low sampling intervals. Our results highlight the difficulty of comparing model results amongst tracking-data sets that vary substantially in temporal grain, and demonstrate the importance of matching the observed temporal resolution of tracking devices to the timescales of behaviours of interest, otherwise inter-individual comparisons of inferred behaviours may be invalid, or important biological information may be obscured.

## Introduction

Understanding the causes and consequences of animal movement and how it is an overt expression of behaviour has become a central theme in biology, especially during the last decade, which has witnessed the birth of the new scientific discipline of ‘movement ecology’ [1]–[3]. Critical to identifying the hierarchy of factors that affect how and why animals move is the choice of methods for characterising their movement patterns [4]. A topic of intense interest to ecologists and ethologists currently is how Bayes' Theorem can be used to infer latent states of behaviour from within individual movement trajectories, or ‘geospatial lifelines’. Examples include stochastic time-series analyses such as state-space models, in which the underlying process represents serial changes in behavioural states [5], and/or the ‘true’ locations of recorded observations [6], [7]. Typically, such models apply Markov-Chain Monte-Carlo (MCMC) fitting techniques to predict distinct modes of behaviour at observed locations, based on information about movement metrics, and less frequently, covariate environmental data [5], [8]. A fundamental assumption of such models is that certain behavioural states (e.g., resting, commuting, area-restricted foraging), because they are the product of quasi-stereotypic responses to movement-inducing stimuli (e.g., food, shelter, mates), can be identified by their geometric configuration in space-time.

Despite the increasing interest, little is known about how the data from which movement trajectories are derived affect the outputs of inferential models. Attributes such as the speed and turning angle between adjacent fixes (as well as more complex measures such as fractal dimension) intrinsically are dependent on the temporal and spatial resolution of sampling regimen. For example, as the interval between sequential location estimates increases, mean rates of movement decrease and turning angles become more uniform [9]. Eventually, temporal grain may become so coarse that biologically meaningful information is lost (at least at fine scales), yet there are practical limits (both physical and computational) to how high the sampling rates of tracking devices can be. Thus, knowledge of how the modelling process and its subsequent outputs are dependent on the temporal resolution of movement data is essential, if the utility of inferential models is to be maximised.

Here, we examine how the sampling interval of animal movement trajectories affects the distributional parameters and states of behaviour inferred by a random-walk movement model (as in Morales et al. [5]). We show results both for a set of synthetically generated data, and for data collected from the common brushtail possum (*Trichosurus vulpecula*), a Phalangerid marsupial native to Australia. Our findings have general implications for comparison of inferred behaviours derived from tracking data sets that differ markedly in temporal resolution. We hope that this paper encourages other researchers to consider how the temporal grain of movement data may influence their modelling results, and to carefully match the sampling interval of tracking devices to study objectives. In demonstrating our findings, we also present several new methods for displaying outputs from MCMC methods that may prove useful to others. An advantage of these methods is that, since model outputs are distributions of parameter estimates (rather than single estimators), outputs are not discretised, but rather are representations of all available information, thus permitting determination of the credible ranges of model parameters.

## Methods

### Generation of synthetic data

We generated a synthetic trajectory of 487 data points (similar in number to that of a possum movement trajectory) made of segments of ‘straight’ and ‘area-restricted’ behaviour. Each straight or foraging segment contained between 20 and 100 data points, chosen at random from a uniform distribution. For each trajectory segment, successive points were generated using the algorithm
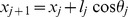


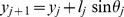
where 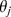 was picked from a Von Mises distribution with mean 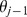 and concentration 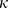 (given by the density function 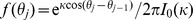 where 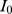 is a modified zeroth-order Bessel function) and 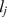 was chosen from a normal distribution with mean 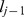 and variance 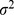.

For the straight segments, 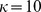 and 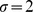. For the area-restricted behaviour, 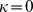 and 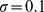. The initial direction of the straight segments was chosen randomly from a uniform distribution. The length of the initial step in a straight segment was 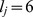 and in an area-restricted segment was 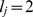. This difference in initial step lengths represents the fact that animals generally move slower during area-restricted movements. Without loss of generality, we choose initial points 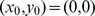 and 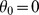.

### Study species and data collection

The common brushtail possum (‘possum’: *Trichosurus vulpecula*) is a primarily folivorous, semi-arboreal member of the marsupial family Phalangeridae. Under normal conditions possums are almost exclusively nocturnal [10], usually emerging from their dens 20–30 min after sunset [11], [12] and often remain active until 1–2 h before sunrise. We choose possums as our study subject because of their high population numbers and ease of capture and handling.

Tracking data for our study were obtained from a population of possums located near Muriwai Beach, on the western coast of the North Island of New Zealand (174.482E, 36.818S). Tracking data modelled in this study were obtained from two male and two female possums between October 2007 and September 2008. We used SirtrackTM (Havelock North, New Zealand) GPS/VHF telemetry collars (*c*. 105 g) to track the nightly movement patterns of the study animals. Collars weighed 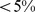 of body mass, and were programmed to determine locations at 5-min intervals, beginning at the approximate time of sunset.

Animals were trapped overnight, fitted with collars and released the following morning, where they were allowed to roam freely for approximately 10 d. To recover the collars, traps were placed around the locations of den sites that were determined during the day by standard radio-telemetry. All capture and handling protocols were approved by the University of Auckland's Animal Ethics Committee (AEC/03/2006/R452).

Location data from the collars were converted from latitude and longitude to the New Zealand Transverse Mercator coordinates using the Blue Marble GeoCalc software (Gardiner, ME). Following standard practice, prior to calculation of inter-fix distances we attempted to remove individual position fixes of questionable accuracy. This was done by applying ‘Option 2’ proposed by Lewis et al. [14], which suggests excluding fixes calculated using information from only three satellites (two-dimensional fixes) that have corresponding values of positional dilution of precision 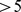. Application of this procedure removed *c*. 10% of the observed fixes.

For additional details on all tracking and handling procedures see Dennis et al. [13].

### Data subsampling and conversion

For both the synthetic and tracking data, observed data consisted of sets of sequential triplets of geographic coordinates and a timestamp 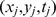, where 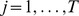 and 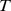 was the total number of observations made.

For the synthetic data, time is in arbitrary units, and data were subsampled at intervals of 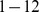 times the original sampling frequency. For each possum dataset, we subsampled the data at intervals of 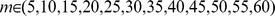 min to simulate the data that might have been obtained had the GPS device recorded a fix every 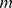 minutes instead of every 5 minutes.

Data were converted to movement rates 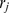 and turning angles 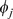 using the formulae:
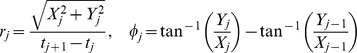
where 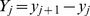 and 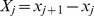 and the 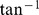 function was computed so as to take account of the quadrant of the argument and give a solution in the range 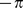 to 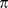.

Because in this study we are investigating the effects of sampling rate on model fitting, it was necessary to ensure that within a given trajectory the data were sampled at fixed frequencies. To reduce the effects of missing data in the possum data, if data points required to compute a given value of 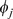 or 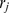 were not available, then that value of 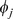 or 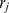 was excluded from further analysis. The sub-sampling algorithm used is described in Appendix S1. Note that the set of locations used at higher sampling intervals was not necessarily a subset of those used for lower sampling intervals. That is, data used for 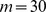 may contain position fixes that were not used for 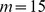.

### The models

To evaluate the effect of the temporal grain of movement trajectories on model outputs we used a multiple-random-walk model first introduced by Morales et al. [5]. Movement paths of individuals were assumed to be comprised of steps and turning angles, with distributions of these attributes specified by one of a set of correlated random walks. We considered models with one, two or three behavioural states. In each model, each observation of a trajectory is classified as belonging to a particular behavioural state, which corresponded to a different random walk. The parameters in the models include both the parameters specifying the speed and turning angle distributions, as well as the state to which each observation is assigned.

For a data set of movement rates 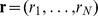, and turning angles 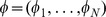, the likelihood of observing the data is given by the density function
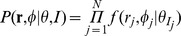
where 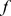 is a probability density function for 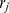 and 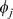 at one step in the time series of data, 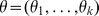 is a vector of parameters, where 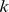 is specified and is the number of different behavioural states in the model. Each 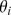 is a vector of parameters describing the random walk model in each of the 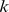 behavioural states. The vector 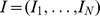 describes the behavioral state at each timestep, and each 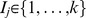. In this paper, we do not model the switching behavior between states directly, that is, each state 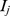 is determined independently (as described below). Including models of switching behavior is a simple extension of the model we use, and is described in detail by Morales et al. [5].

Following Morales et al. [5], we use
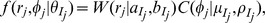
where
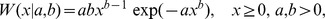
is a Weibull distribution and
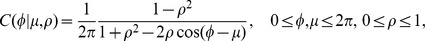
is a wrapped Cauchy distribution. Thus for each 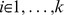, the vector 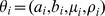 are the parameters describing the random walk in each behavioral state.

The Weibull distribution has a mean of 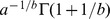, and a mode of 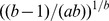 if 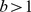, and a zero mode otherwise. The mean of the wrapped Cauchy is 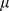, and 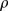 is the concentration parameter. As 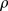 approaches one, the mean vector tends to a point on the unit circle, that is, the distribution converges to a point distribution at 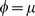. As 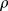 approaches zero, the mean vector tends to the origin, and the distribution converges to a uniform distribution on 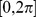.

We considered three models, which differed only by the maximum number of behavioural states that were allowed. That is, we considered the three cases 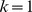, 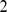, or 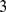. In each model, the vector of all parameters is 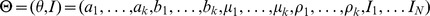. For each model, parameters were fitted to the data by MCMC techniques, using the software WinBUGS (Lunn et al. 2000 [15]). WinBUGS creates a chain of samples of the parameters 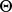, using Gibbs sampling to update from the sample 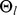 to the sample 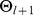.

Vague priors were used wherever possible, specifically, for the parameters 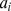 and 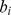 we used Gamma distributions with means of 30 and variances of 3000. For the parameter 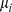 the prior was uniform from 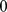 to 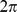 and for the parameter 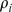 the prior was a uniform distribution which ranged in value between 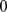 and 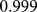 (we require a maximum 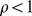 so that we can define a maximum value for 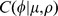 in the WinBUGS code).

For each model we ran a single MCMC chain for 100,000 iterations (after a burn-in period of 2000 iterations), keeping every 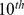 MCMC sample of the parameters 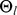 for posterior estimation. Convergence of the chains was checked for a random sample of parameters. In mixture models such as our two- and three-state variants, posterior distributions are symmetric with respect to permutations of the labels of the states. This causes ‘label-switching’ within the Markov Chain. We used the re-labelling algorithm of Stephens [16] to address this problem before performing any posterior analyses.

For the synthetic data, we used MCMC methods to fit the two-state model at each of the twelve sub-sampling intervals. For each of the four possums, we used MCMC to fit all three models, at each of the twelve sub-sampling intervals.

### Visualisation techniques

The output of the MCMC model-fitting techniques contains a wealth of information, and in the Results section we present several novel ways of displaying such data. The methodology described here could be used for the displaying the output of any fitting process using MCMC methods.

A standard method of displaying results of MCMC fitting procedures is to show posterior distributions of the parameters, in this case 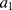, 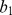 etc. However, in our situation, distributions of these parameters do not give particularly useful information about movement rates or turning angles. Instead, we compute what we term ‘posterior distributions’.

For example, we label the parameter sample from the MCMC fitting procedure as 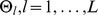, where in this case 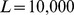. To compute the posterior Weibull distribution for behavioral state 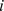, we use
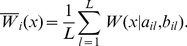
(1)Note that since 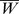 is the mean of a set of distributions, the area under 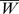 will also integrate to one. The mean of a Weibull distribution is 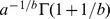, so we define the posterior mean of the Weibull distribution to be
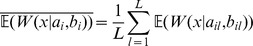
In a similar fashion, we compute the posterior wrapped Cauchy distribution, and the posterior mean vector for the wrapped Cauchy distribution. Variances of the posterior means also can be determined, relative to the MCMC samples.

**Figure 1:**
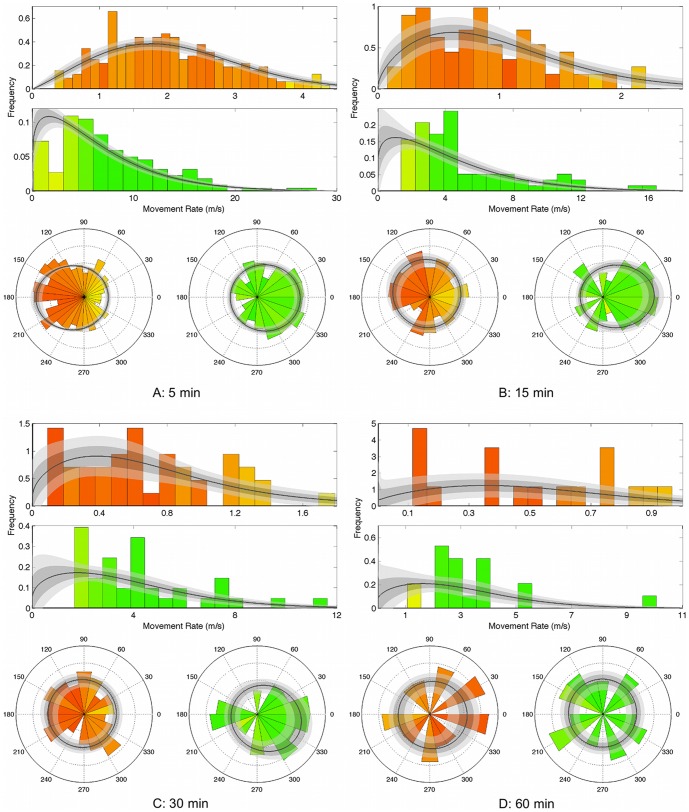
Movement-rate and turning-angle distributions of one set of trajectories (possum #1882). Each sub-figure shows, for each state in the two-state model, movement-rate (top, in m/s) and turning-angle (bottom, in degrees) distributions, at subsampling frequencies of (a) 5 min, (b) 15 min, (c) 30 min and (d) 60 min. Lines and colors are as in Fig. 2.

**Figure 2:**
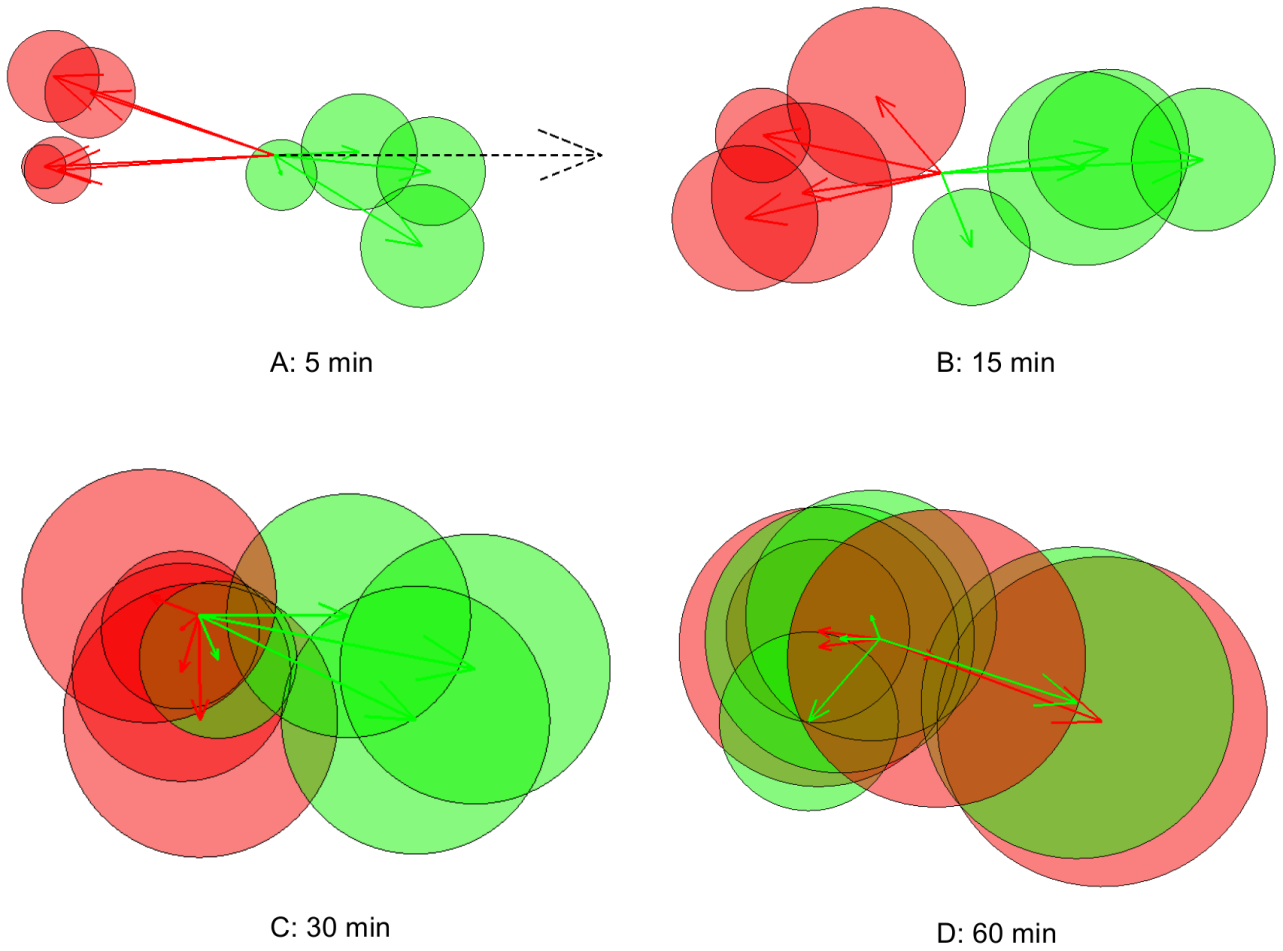
Mean vectors of posterior turning-angle distributions. Mean vectors are represented for subsampling intervals of 5, 15, 30 and 60 min, for both behavioural modes (red is State 1, green is State 2), for each of the four possums. Each arrow and circle represents one behavioural mode for one possums. Circles around arrow heads have radii equal to the posterior standard deviation of mean vectors. The dashed arrow in (a) is of length 0.4 (the maximum possible length for a mean vector is 1, which corresponds to a point distribution).

**Figure 3:**
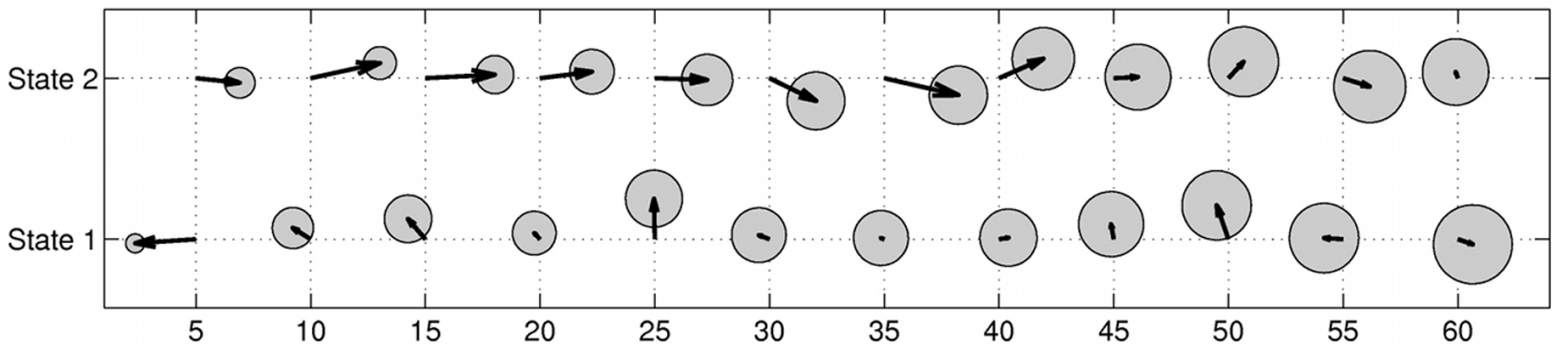
Posterior mean vectors of turning-angle distributions of possum #1882. The figure shows the posterior mean vectors of the wrapped Cauchy distribution of turning angles, for each sub-sampling interval and behavioural state. Circles around each arrow head have radius equal to the posterior standard deviation of the mean vector.

In generating the posterior distributions in this manner, we were able to use all the information from the MCMC output. Examples of this approach are in Fig. 1, showing posterior movement rate and turning angle distributions. In this figure, we also indicate the confidence of the posterior mean distributions, by plotting the curves obtained by taking one or two standard deviation either side of the values computed in equation (1) (with respect to the MCMC samples). These curves are shown by the grey shaded areas. Note that these curves do not represent distributions, but if the standard deviations are large, we have less confidence in the results of the MCMC fitting procedure than if they are small, and hence the curves contain useful information. Figs. 2 and 3 show posterior means and variances of the mean vector of the wrapped Cauchy distribution.

**Figure 4:**
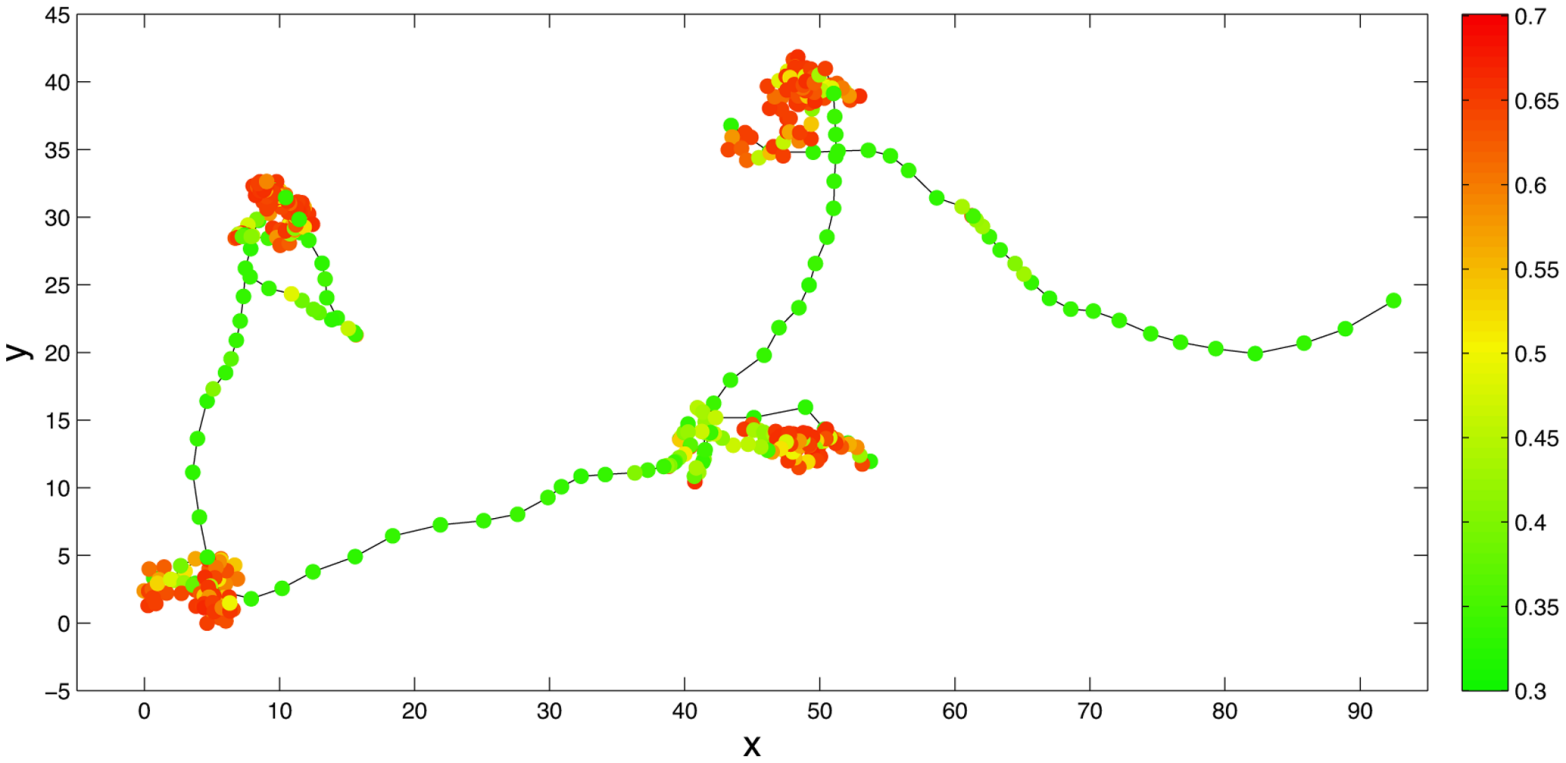
Synthetic data, at original frequency. Inferred behavioural states from the MCMC fitting are shown for each location (red is ‘slow-area-restricted’, green is ‘fast-directed’), and the colour scale indicates the posterior probability of each data point being in State 1.

We also use the information from the posterior distributions of the behavioral state parameters 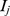 in displaying our results. For each 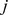, we take the mean over the 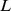 samples to create a posterior probability for each of the trajectory's data points belonging to a particular behavioural state. Rather than discretising the output, and assigning each data point to one or other of the states, we represent this probability by a continuous colour gradient. An example of this can be seen clearly in Fig. 4, where each data point is coloured with respect to its probability of being in State 1. We also use this method for the histogram plots in Fig. 1, where each bar is coloured with respect to the mean probability for the states ‘counted’ in that bar.

These methods provide a simple way of displaying more details of the model outputs, and allow for easier recognition of the biological signals ‘hidden’ in the data.

## Results

### Results from synthethic data

The synthetic position data are shown in Fig. 4. The two-state model divided the data into two distinct modes, characterised primarily by differences in movement rates. We refer to the ‘slow-area-restricted’ state as ‘State 1’ and the ‘fast-transiting’ state as ‘State 2’. Results from the MCMC fitting for the data at the original resolution are also shown in Fig. 4, with a colour scale from red to green indicating the posterior probability of that location being in State 1 (red) or State 2 (green). The MCMC model correctly classifies 86% of the points.

**Figure 5:**
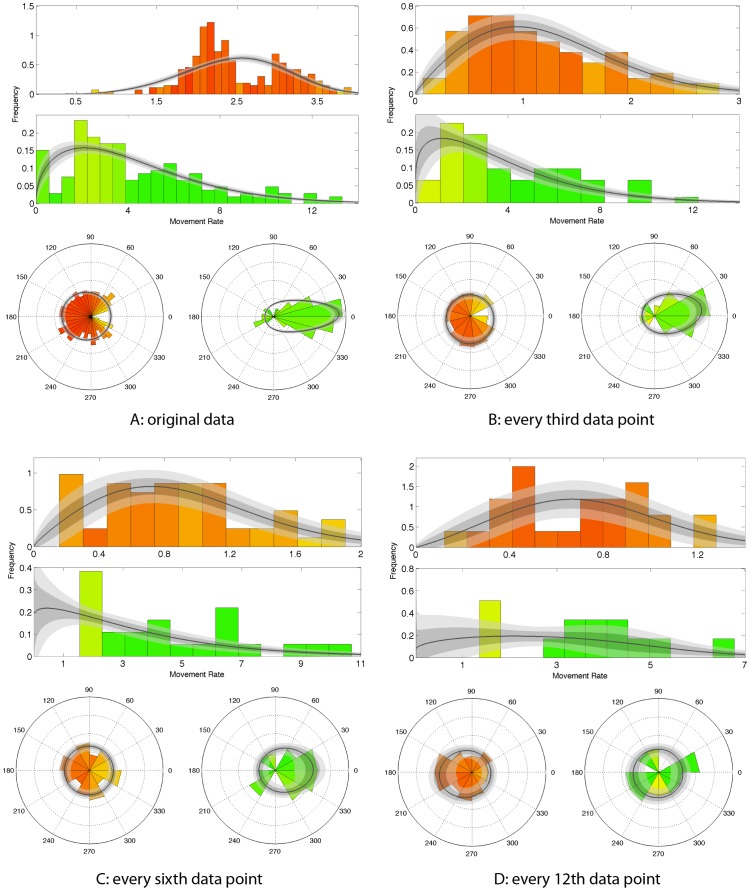
Movement-rate and turning-angle distributions for MCMC output from synthetic data. Each sub-figure shows, for each state in the two-state model, movement-rate (top) and turning-angle (bottom) distributions, at subsampling frequencies of (a) 1, (b) 3, (c) 6 and (d) 12 units, where the original data are ‘observed’ every unit. Solid lines are posterior Weibull and wrapped Cauchy distributions, with 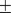 one (two) posterior standard deviations shown in dark (light) grey. Histograms are observed frequencies, with division of data into states using output from MCMC computations. Histogram bars are coloured according to the mean probability of observations in that bar being in State 1 or 2, as given in the colour bar in Fig. 1, that is, red bars are more likely to be in State 1 and green bars more likely to be in State 2. Yellow bars are close to being undetermined - that is, they have an approximately equal probability of being in either state.

Fig. 5 shows the results of the MCMC fitting procedure for the thinned data. The turning angles and movement rate distributions are shown for each state. Solid lines indicate posterior Weibull and wrapped Cauchy distributions, with 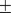 one (two) posterior standard deviations shown in dark (light) grey. The histograms represent the frequencies observed from the data with a greater-than-50% probability of being in that state, with each bar coloured according to the mean posterior probability that data points in that interval should be classed in that particular state. The distinction between slow and fast movement rates between states at all sub-sampling frequencies is clear. For the original data, State 1 has a turning-angle distribution which has a peak around 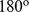 while State 2 has a distribution which is highly peaked around 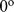, as is the case in the algorithm generating the data. As the frequency between data points decreases, both turning angle distributions become more uniform.

### Results from possum trajectories

From the 36 individual tracking nights that were modelled in this study we obtained a total of 3272 location estimates (mean of 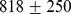 *SE* fixes per animal). It should be noted that the GPS collars only recorded locations when the possums were outside of dens, where it was possible to receive data from satellites. Thus, the number of locations obtained was lower than what would be expected had the collars collected data continuously during the 11-h operational period, because periods of activity generally ranged between only 6 and 9 h.

Tables of the estimated parameter values from the MCMC output for all possums are available in the supplementary material. We first consider in detail the results from the two-state model, which clearly show the differences in posterior distributions that occur when the sub-sampling interval increases. We then briefly discuss comparisons with the single and three-state models.

### Results from the two-state model

As for the results from the synthetic data, we refer to the ‘slow-area-restricted’ state as ‘State 1’ and the ‘fast-transiting’ state as ‘State 2’. We first discuss some general results for all possums, and then to show greater detail, provide more specific results of a single animal (#1882). For low values of 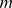 (approximately 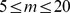), State 1 is characterised by high mean-turning angles and low movement rates, while conversely, State 2 is associated with low mean-turning angles and high movement rates. We consider these states to correspond with ‘area-restricted’ and ‘transiting’ modes of behaviour, respectively. For higher values of 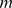 (approximately 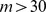), both states have turning-angle distributions that are approximately uniform over all angles.

**Figure 6:**
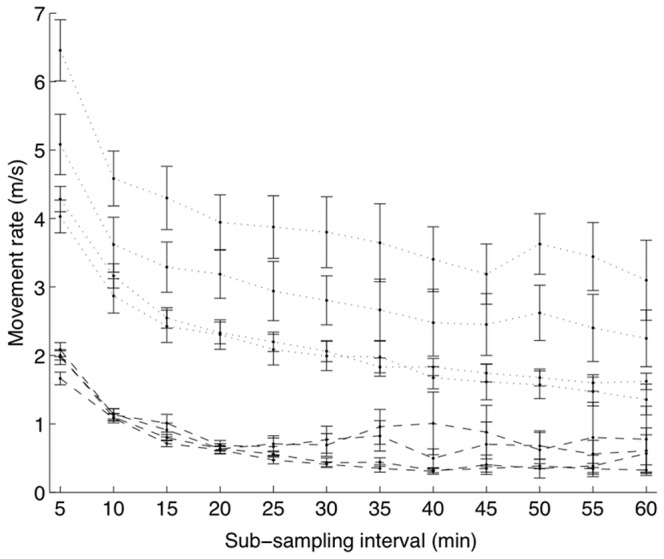
Posterior means of movement-rate distributions with respect to the temporal resolution of modelled trajectories. The figures show the posterior means of the Weibull distributions for movement rates (in m/s), with error bars of one posterior standard deviation, for each of the four possums, as a function of sub-sampling interval. State 1 is indicated with dashed lines, and State 2 with dotted lines. As expected, the movement rates decrease with increasing sub-sampling interval.

As expected, mean movement rates in both states decreased with increasing sub-sampling interval (Fig. 6). It can be seen (Fig. 2) that for the smaller sub-sampling intervals (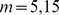), State 1 has a mean turning-angle vector close to the negative real axis, meaning a high mean turning angle with a peaked (‘leptokurtic’) distribution. Similarly, State 2 is characterised by a mean turning-angle vector close to the positive real axis, indicating a low mean turning angle, again with a leptokurtic distribution. As sub-sampling intervals increased, the mean vectors for both states approached zero, and the posterior variance of the mean angles increased (Fig. 2), suggesting that a uniform distribution of turning angles was more likely.

**Figure 7:**
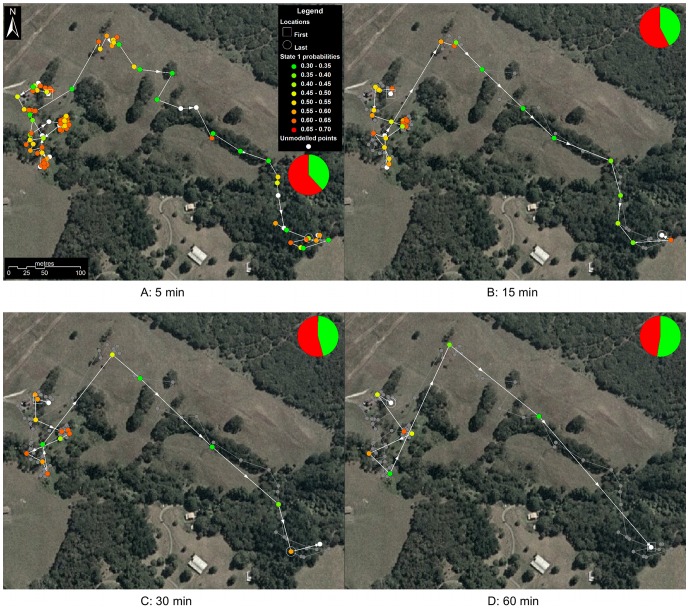
Data from a single night's activity of possum #1882. Example of a nightly movement trajectory of possum #1882 at sub-sampling intervals of 5, 15, 30 and 60 min. Inferred behavioural states are shown for each location estimated by the two-state model. Panel (a) shows the original trajectory as recorded at 5-min intervals from a GPS collar. Panels (b–d) indicate sub-samples of the original trajectory at progressively larger intervals (15, 30, and 60 min, respectively). Piecharts show the proportions of the two behavioural modes (red is ‘slow-area-restricted’, green is ‘fast-directed’). In this example, it is obvious that as sub-sampling interval increases, the proportion of ‘slow-area-restricted’ behaviour correspondingly decreases (by 25%). The figure also demonstrates the considerable loss of information about behaviour at larger sampling intervals.

Due to space limitations, we present detailed results only for one individual (#1882). Figs. 1 and 7 represent the data only from sub-sampling intervals of 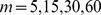.

Fig. 1 shows the turning angles and movement rate distributions for each state, for possum #1882. as for the synthetic data shown in Fig. 5. The distinction between slow and fast movement rates between states at all sub-sampling frequencies is clear. For 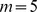 and 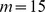, State 1 has a turning-angle distribution which is centred and peaked around 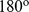. Additionally, turning angles further from 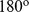 are those that are less likely to be considered to be representative of State 1. Mean turning angles for State 2 are near zero. Conversely, for 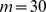 and 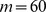, turning-angle distributions for both states are approximately uniform.

In Fig. 7 we show the data from a single night's activity, with state probabilities again represented by colour, over an aerial photograph of the study site.

Fig. 3 shows the posterior-mean vectors for the angular distributions over all sub-sampling intervals. For small 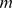, the mean turning angle vector for State 2 (‘fast-transiting’) is near the positive real axis, therefore the mean turning angle is small and the distribution is peaked around that point. It can be seen that the variance of the mean vector increases as the sub-sampling interval increases. The mean turning angle vector for State 1 (‘slow-area-restricted’) is close to the negative real axis for small sub-sampling intervals. This corresponds to a high mean-turning angle and a peaked distribution. As the sub-sampling interval increases, the magnitude of the mean vector decreases, again demonstrating that the turning-angle distribution becomes more uniform.

### Comparison with single-state and three-state models

While it is not our intention in this paper to find the ‘best’ model that fits the observed data, we also apply one and three-state models to the possum tracking data to examine relationships among the numbers of states and sampling intervals. In this section we briefly discuss the results of this investigation.

For the one-state model, obviously there is no division of data into different modes. In this model the posterior variances of both movement rates and turning angles increased as 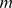 increased. The turning-angle distribution tended to be uniform even for small 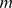.

In the three-state model, many of the same phenomena that were observed in the two-state model were also evident as the sampling interval increased. The three states were, for small 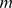, typically (i) fast, with a small turning angle, (ii) slow with a large turning angle, and (iii) slow with a uniformly distributed turning angle. As 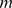 increases, the turning angle distributions of all three states becomes more uniform.

## Discussion

We have shown that the temporal grain of movement trajectories has profound influence on the observed distributions of posterior model parameters, and thereby on the states of behaviour inferred by a movement model. To our knowledge, our study is the first systematic assessment of such effects that has been published in the primary literature. Although we have demonstrated our findings using location data obtained from GPS devices, the results of our study also are relevant for data obtained by other remote-tracking methods, such as radiotelemetry, ARGOS satellite telemetry, or light-based geolocation methods. Differences in sampling intervals and issues of irregularly sampled position fixes are common (although not equivalent) amongst these data types [17].

Determination of how the temporal resolution of tracking data affects the parameterisation of inferential movement models is a complex issue, given the many factors that influence the modelling process. Multifarious interactions among these factors, which include the choice of behavioural template, the metrics used to characterise movement, and biological scales of interest make it difficult to draw general conclusions regarding their hierarchical importance. In this paper we did not systematically consider differences between observation- and process-related scales (see Bovet & Benhamou, 1988 [19]; Benhamou, 2004 [20], Codling & Hill, 2005 [9]), or their influence on the nature of information that can be derived from inferential movement models. From a pragmatic perspective, because such models are becoming increasingly popular (Patterson et al., 2008), it is essential that researchers who employ these methods are aware of the various factors that affect the modelling progression. While knowledge of the influence of observation- and process-related scales is of great importance, and most definitely should be methodically addressed in subsequent work, a formal treatment of this matter is beyond the scope of our paper. We suggest that effects of temporal grain should be considered throughout all phases of the modelling process, from how it influences measured attributes of movement, to how movements subsequently are expressed as behaviours. Failure to do so may lead to poor representation of the biological patterns of interest, and ultimately, wasted research efforts.

### Possum movement model

In this paper we considered a two-state behavioural model and examined changes in the distributions of turning angles and movement rates for each of the two random walks. In all cases, the two-state model produced both ‘fast’ and ‘slow’ states. For high-subsampling frequencies, the slow state had a turning-angle distribution that was peaked around 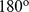, while the fast state had a turning-angle distribution that was peaked around 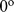; our most important finding is that as the sampling interval increased, the distribution of turning angles of both states became more uniform. This result held for both synthetic and empirical data. It is obvious in the original 5-min trajectories (in the possum data) that locations classified in the ‘slow’ state, because of high turning-angles and slow speeds, are associated with ‘area-restricted behaviour’ in which the possum either foraged or rested in a localised area. However, it is much less clear what the turning-angle distributions of lower-temporal-resolution trajectories represent biologically. Turning-angle distributions of fast states also changed, although less significantly in the possum tracking data than the synthetic data. For high sampling frequencies, turning-angle distributions of fast states were peaked around zero, indicating a ‘transiting’ or ‘commuting’ mode of behaviour. Similar to the slow state, turning-angle distributions of fast states became more uniform as the sampling frequency decreased, and again it is less apparent what this state represents biologically.

One possible criticism of the use of Bayesian methods in this manner is that the data sets at lower resolution naturally contain fewer data points, and in a Bayesian analysis, this means that the posterior distribution will look more like the prior distribution, which for the turning angles, was uniform. However, we believe that the number of data points used, even for the 60-min-interval data set, was sufficiently large for the prior distribution to have a very limited effect on the posterior distributions. To test this, we repeated the MCMC fitting procedure for a subset of the 5- min data set for possum #1882, such that the subset contained the same number of data points as the 60-min data set. Appendix S2 contains the results of this analysis.

It is abundantly clear that as the temporal resolution of movement trajectories decreases, information about behaviour also decreases. Codling and Hill [9] studied the effects of sampling rate on various types of random walks. They showed that as sampling frequency decreased, observed movement rates also decreased, because of ‘smoothing effects’ and reduction in temporal autocorrelation, which tends to make turning-angle distributions more uniform. These same effects also are inherent in our multi-state models: mean speeds of both ‘slow’ and ‘fast’ states decreased as sampling frequencies decreased, and turning-angle distributions became more uniform. Moreover, we also observed higher posterior variances in distributions as the temporal resolution of trajectories decreased.

We must additionally consider how variation in temporal resolution affects the division of trajectories into multiple states. Specifically, for high frequency trajectories, we were able to discern that high turning-angles were correlated with low movement rates (area-restricted behaviour), and that low turning angles were correlated with high rates of movement (transiting behaviour). However, because the turning-angle distributions of all states became more similar as sampling rates decreased, correlation was no longer apparent and the ability to distinguish different states of behaviour was lost. For the possums in our study, a 5-to-15 min sampling interval was sufficient to detect the typical nightly pattern of two-to-three sessions of feeding behaviour at two-to-four different sites. These were usually separated by several hours of comparative inactivity, and collectively sum to about 45% of the *c.* 8 h possums spend outside of their dens [11], [12], [18].

We did not attempt to identify the precise sampling frequency at which changes in inferred behaviours were related to temporal resolution. However, we note that to successfully discriminate distinct behavioural modes, the sampling frequency of trajectory data must be high enough so that there are sufficient location observations recorded during periods of each state to detect differences in movement patterns.

### Practical implications

Our study highlights two important considerations when modelling behaviour using movement data. First, researchers should match the sampling intervals of tracking devices to the approximate time scales of behaviours of interest. For example, if the principal objective of a hypothetical study of common brushtail possums were to elucidate the relationships between foraging bouts and micro-habitat structure, then GPS collars configured to record location estimates at 5-min intervals likely would provide sufficient data with which to identify even single trees as important landscape elements. Successful discrimination of the importance of single landscape features probably would not be possible if movement trajectories were characterised at much lower temporal granularities (e.g., 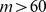 min), especially if such features were visited only infrequently, or the average duration of visits was 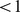 h. Conversely, if the primary aim were description of behaviours occurring over much longer time scales (e.g., long-distance dispersal, or extra-range excursions that last on the order of days), then 60-min sampling intervals most probably would suffice. A more extreme example is the difference between the temporal scales of the diurnal movement patterns of seabirds (for example, foraging trips during chick rearing) and the transit phase of annual migratory events, which can last for weeks (e.g., Egevang et al. [21]). Because of the trade-offs between sample frequency and operational life that are inherent to satellite-telemetry devices [22]–[24], it may not be possible to simultaneously model behaviours that occur over vastly different time scales using location data obtained by these technologies. Concurrent deployment of tracking devices configured to record location observations at markedly different sampling intervals on different individuals may be an efficient means of simultaneously collecting data with which to model behaviour over multiple temporal scales.

A second important consideration when modelling behaviour within movement data is that the temporal resolution of trajectories should be broadly similar if inter-subject or inter-study comparisons are to be valid. We emphasise that both the configured and observed (i.e., that actually obtained and used following data pre-processing) sampling rates of data-capture devices affect the temporal grain of geospatial lifelines. This being the case, both attributes jointly will influence the ability of movement models to discriminate behavioural modes. It is well known by users that the performance characteristics of currently available remote-tracking technologies are dependent at least to some extent on aspects of both the environment and behaviour of study subjects. For example, the accuracy and fix-success rates of GPS receivers are strongly modified by topography and vegetation [25]–[27], as well as by body orientation [28] and patterns of activity [29], [30]. Likewise, environmental and species-specific factors are reported to affect the performance of ARGOS PTTs and light-based geolocators [31]–[33]. Another important factor affecting the temporal resolution of movement trajectories is the choice of method(s) by which researchers attempt to remove fixes with large location errors from positional data sets prior to modelling. (State-space models incorporating observation error do not require this.) Depending on the tracking technology, the screening method, and the willingness of researchers to retain error-prone data, such procedures can exclude substantial numbers of observed locations (e.g., 35% of the fixes of a black bear data set using ‘Option 4’ in Lewis et al. 2007 [14]), with obvious implications for the temporal grain of trajectories. We found in our study that there was a clear bias between the sampling interval of trajectories and the proportions of inferred behavioural states. To correctly make inter-individual comparisons, it may be necessary to re-sample some trajectories so that temporal grain is standardised. Failure to account for differences in temporal resolution may lead to biased or erroneous inferences about behaviour.

In light of our findings, we suggest researchers tailor their sampling regimes to account for limitations in data-collection methods, and develop objective means of validating model parameters, to ensure that inferred states of movement are representative of ‘real’ behavioural patterns. We also recommend that whenever possible pilot studies be conducted on a sample of test animals to assess how well data-capture devices operate at study sites and to help determine which sampling intervals (if variable configurations are possible) are most appropriate for research objectives. In some instances, it may be prudent to configure tracking devices to record location estimates at the highest sampling frequency that is logistically feasible (i.e., ‘over sample’), given the constraints of body size, battery life of tracking devices, and study aims. High sampling rates will help compensate for lost data and also may provide sufficient information with which to detect short-lived, or ‘spatially indistinct’ behaviours that may be infrequent but important elements of life cycles.

## Conclusions

Inferential movement models provide an effective framework for classifying observer-free patterns of behaviour within the geospatial lifelines of animals. The utility of these models may be further enhanced through association of predicted behaviors with corresponding information about physiological states or environmental conditions (see reviews in Cooke et al. [34], Patterson et al. [7], Schick et al. [3]). Despite the potential dangers of complex models, the popularity and value of such methods almost certainly will increase as fine-scale movement trajectories become easier to obtain (due to reductions in the size, cost, and spatial error of tracking devices), as the computational power of computers improves, and as modelling algorithms become more mathematically tractable (because of advances in the functionality and ease-of-use of modelling software). As a new paradigm for study of movement phenomena emerges, there will be increased interest in modeling the dynamic processes that influence the movement and space-use of animals. Understanding how the temporal grain of re-location data affects the functionality and validity of such models thus is of paramount importance.

## Supporting Information

Table S1
**Table showing posterior parameter values for possum** 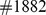 **for all three models, and each sub-sampling interval.**
(PDF)

Appendix S1 **Appendix describing sub-sampling algorithm.**
(PDF)

Appendix S2
**Appendix repeating MCMC fitting procedure on a subset of the 5 min data for possum** 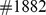 **.**
(PDF)
