# Supplementary material for: Effects of Temporal Resolution on an Inferential Model of Animal Movement
Source: PLoS One. 2013 May 6;8(5):e57640. doi: 10.1371/journal.pone.0057640 (PMC3646004; doi:10.1371/journal.pone.0057640)
Supplement: Table S1 — Table showing posterior parameter values for possum for all three models, and each sub-sampling interval. (PDF) [file pone.0057640.s001.pdf]

Table S1

Mean posterior parameter values for possum #1882 for all three models, and each sub-sampling interval.

		5	10	15	20	25	30	35	40	45	50	55	60
Single-state model	a_1	0.20	0.34	0.40	0.44	0.46	0.46	0.50	0.58	0.51	0.45	0.53	0.52
	b_1	1.11	1.04	0.97	0.99	0.95	0.97	0.95	0.90	0.99	1.05	0.95	1.00
	ρ_1	0.06	0.15	0.15	0.13	0.17	0.11	0.18	0.19	0.10	0.18	0.09	0.09
	μ_1	2.84	0.39	0.42	0.33	0.68	-0.56	-0.17	0.42	0.70	1.54	-0.31	0.20
Two-state model	a_1	0.16	0.66	0.85	1.71	1.78	1.42	1.30	2.63	1.78	1.77	2.30	2.70
	a_2	0.10	0.11	0.16	0.11	0.12	0.14	0.15	0.12	0.10	0.05	0.09	0.18
	b_1	2.16	1.82	1.63	1.94	1.58	1.50	1.22	1.37	1.12	1.50	1.40	1.73
	b_2	1.22	1.38	1.23	1.60	1.50	1.45	1.51	1.72	2.03	2.60	2.10	1.54
	μ_1	-3.07	2.58	2.25	2.33	1.63	2.76	2.74	0.18	1.75	1.90	3.08	-0.37
	μ_2	-0.10	0.21	0.05	0.12	-0.03	-0.46	-0.22	0.43	0.08	0.85	-0.27	1.95
	ρ_1	0.33	0.13	0.16	0.09	0.24	0.12	0.11	0.13	0.15	0.23	0.17	0.18
	ρ_2	0.23	0.40	0.39	0.29	0.29	0.30	0.44	0.29	0.18	0.19	0.20	0.14
Three-state model	a_1	0.25	0.74	2.05	21.49	28.40	3.77	9.33	11.71	14.98	41.10	9.69	19.33
	a_2	0.07	0.25	0.41	0.72	0.49	0.63	0.43	1.26	1.02	1.16	1.42	5.83
	a_3	0.10	0.07	0.15	0.11	0.29	0.13	0.08	0.09	0.10	0.04	0.07	0.17
	b_1	2.55	1.99	1.98	3.52	3.50	1.88	1.89	1.95	1.59	2.22	1.58	2.04
	b_2	3.00	1.58	2.06	3.13	1.91	1.73	2.20	1.67	4.26	4.14	6.09	5.44
	b_3	1.23	1.71	1.28	1.58	1.43	1.59	2.13	2.44	2.08	2.73	2.55	1.75
	μ_1	2.89	2.32	1.74	2.94	2.90	-2.67	-1.80	-0.98	2.02	2.20	2.71	-0.14
	μ_2	2.87	-0.26	2.28	0.32	0.54	2.73	1.43	1.15	0.25	1.84	-3.10	-3.14
	μ_3	-0.27	0.30	0.03	0.10	0.42	-0.42	-0.22	0.22	0.19	0.70	-0.21	1.65
	ρ_1	0.21	0.13	0.17	0.21	0.22	0.20	0.18	0.21	0.21	0.19	0.19	0.40
	ρ_2	0.49	0.21	0.30	0.19	0.48	0.25	0.17	0.27	0.22	0.38	0.31	0.30
	ρ_3	0.25	0.52	0.42	0.29	0.30	0.35	0.55	0.36	0.18	0.21	0.23	0.15
