# Supplementary material for: Effects of Temporal Resolution on an Inferential Model of Animal Movement
Source: PLoS One. 2013 May 6;8(5):e57640. doi: 10.1371/journal.pone.0057640 (PMC3646004; doi:10.1371/journal.pone.0057640)
Supplement: Appendix S1 — Appendix describing sub-sampling algorithm. (PDF) [file pone.0057640.s002.pdf]

Appendix S1

In this appendix, we describe the algorithm which is used to sub-sample the trajectories. For each dataset, we used intervals of $m \in (5, 10, 15, 20, 25, 30, 35, 40, 45, 50, 55, 60)$, and a maximum time difference between successive points of $p = 1.5m$.

Sub-sampling algorithm:

1. For i=1 to T, find dt(i)=t(i+1)-t(i). If dt(i)<m-2.5, then discard point (i+1). Repeat until dt(i)$\geq$ m-2.5.

2. (a) If dt(i)$\leq$ p, keep data point (i+1) and return to 1.

   (b) If dt(i)>p, then the dataset from points 1 to i forms a sub-trajectory. Store sub-trajectory and remove from the dataset, return to 1.
