# Supplementary material for: Effects of Temporal Resolution on an Inferential Model of Animal Movement
Source: PLoS One. 2013 May 6;8(5):e57640. doi: 10.1371/journal.pone.0057640 (PMC3646004; doi:10.1371/journal.pone.0057640)
Supplement: Appendix S2 — Appendix repeating MCMC fitting procedure on a subset of the 5 min data for possum . (PDF) [file pone.0057640.s003.pdf]

Appendix S2

We repeated the MCMC fitting procedure for a subset of the 5 min data set for possum #1882, such that the subset contained the same number of data points as the 60 min data set (49). We display the results in the same format as used in figure 5 in figure S1. Comparison of this figure with those in figure 5 shows that for this subset of 5 min data, the turning angle distributions of both the slow and fast states are peaked, as is the case when using the full 5 min data set. Comparison of this result with those for the 60 min data set makes it clear that the small number of data points does not imply that the posterior distributions of the turning angles will be uniform.

Figure S1

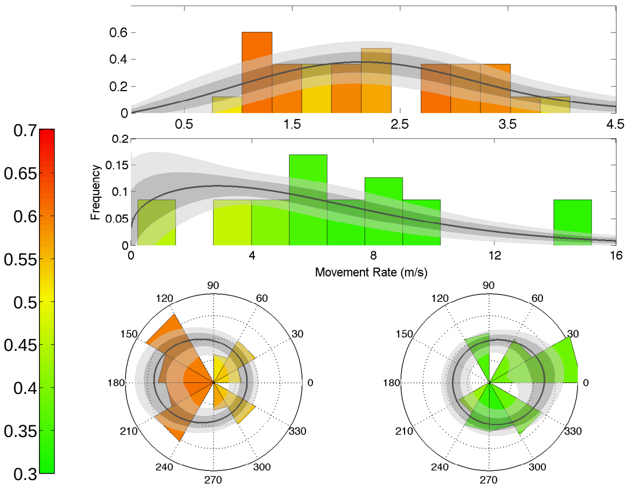

Movement-rate and turning-angle distributions of one set of trajectories (possum #1882). The figure shows, for each state in the two-state model, movement-rate (top, in m/s) and turning-angle (bottom, in degrees) distributions, for a subset of the data with frequency of 5 min. Lines and colours are as in figure 2.
